# QTL Detection for Rice Grain Length and Fine Mapping of a Novel Locus *qGL6.1*

**DOI:** 10.1186/s12284-022-00606-z

**Published:** 2022-11-28

**Authors:** Mingzhu Zhao, Yuanzheng Wang, Na He, Xiu Pang, Lili Wang, Zuobin Ma, Zhiqiang Tang, Hong Gao, Liying Zhang, Liang Fu, Changhua Wang, Jingang Liu, Wenjing Zheng

**Affiliations:** 1 Institute of Rice Research, Liaoning Academy of Agricultural Sciences, Shenyang, 110000 China; 2 Institute of Crop Research, Liaoning Academy of Agricultural Sciences, Shenyang, 110161 China

**Keywords:** Rice, Grain length, QTLs, Fine mapping, Candidate genes

## Abstract

**Background:**

Grain length (GL) that is directly associated with appearance quality is a key target of selection in rice breeding. Although abundant quantitative trait locus (QTL) associated with GL have been identified, it was still relatively weak to identify QTL for GL from *japonica* genetic background, as the shortage of *japonica* germplasms with long grains. We performed QTLs analysis for GL using a recombinant inbred lines (RILs) population derived from the cross between *japonica* variety GY8 (short grains) and LX1 (long grains) in four environments.

**Results:**

A total of 197 RILs were genotyped with 285 polymorphic SNP markers. Three QTLs *qGL5.3*, *qGL6.1* and *qGL11* were detected to control GL by individual environmental analyses and multi-environment joint analysis. Of these, a major-effect and stable QTL *qGL6.1* was identified to be a novel QTL, and its LX1 allele had a positive effect on GL. For fine-mapping *qGL6.1*, a BC_1_F_2_ population consisting of 2,487 individuals was developed from a backcross between GY8 and R176, one line with long grain. Eight key informative recombinants were identified by nine kompetitive allele specific PCR (KASP) markers. By analyzing key recombinants, the *qGL6.1* locus was narrowed down to a 40.41 kb genomic interval on chromosome 6. One candidate gene *LOC_Os06g43304.1* encoding cytochrome P450 (CYP71D55) was finally selected based on the difference in the transcriptional expression and variations in its upstream and downstream region.

**Conclusions:**

Three QTLs *qGL5.3*, *qGL6.1* and *qGL11* were identified to control grain length in rice. One novel QTL *qGL6.1* was fine mapped within 40.41 kb region, and *LOC_Os06g43304.1* encoding cytochrome P450 (CYP71D55) may be its candidate gene. We propose that the further cloning of the *qGL6.1* will facilitate improving appearance quality in *japonica* varieties.

**Supplementary Information:**

The online version contains supplementary material available at 10.1186/s12284-022-00606-z.

## Introduction

Grain length (GL) is the main factor that determines appearance in rice, and affect milling, cooking and eating quality, and is therefore an important agronomic traits in rice (*Oryza sativa* L.) breeding. (Fitzgerald et al. 2009). The *indica* varieties generally exhibit long grains, while the *japonica* varieties have short grains. However, with the wide popularity of some *japonica* varieties with long grains, such as Daohuaxiang, among the consumers, increasing GL has become an important target in *japonica* breeding recently (Huang and Qian 2017).

As rice GL is quantitatively inherited (McKenzie and Rutger 1983), it is difficult for breeders to efficiently improve GL using conventional selection methods. Therefore, it should be particularly beneficial to enhance breeding efficiency to use markers closely related to genes or major quantitative trait loci (QTLs) for GL, while the target genotypes could be screened directly in early generations (Wan et al. 2006). A lot of QTLs associated with rice GL have been detected in the previous studies, and some major or major-effect QTLs as well as their candidate genes have been identified, including *GS3* (Fan et al. 2006), *GS2* (Hu et al. 2015), *GL7* (Wang et al. 2015), *GLW7* (Si et al. 2016), *TGW6* (Ishimaru et al. 2013), *qGL3* (Gao et al. 2019), *GL6* (Wang et al. 2019) and *GW6a* (Song et al. 2015; Gao et al. 2021). Importantly, most of these QTLs or genes were detected from the *indica* varieties with long grains, such as Minghui63 and Kasalath, which were widely used as the long grain parents to cross with the *japonica* to develop the mapping populations. Recently only few QTLs for GL, such as *qGL3.1* and *qGL3.3*, have been identified from *japonica* rice (Hu et al. 2018; Qi et al. 2012; Zhang et al. 2012; Ying et al. 2018; Xia et al. 2018). However, it was still relatively weak to identify QTLs or genes associated with GL from *japonica* genetic background.

To improve GL in *japonica* breeding, it was an efficient way by introgression of alleles from *indica* to *japonica *via subspecies cross. For example, the major QTL *GS3* for grain size was identified from *indica* (Fan et al. 2006), and its long-grain-associated *gs3* allele has been introgressed in *japonica* gene pool by genetic improvement (Sun et al. 2012). Whereas, most of dense and erect panicle *japonica* varieties, released as high-yielding varieties and had extensive production areas in China, did not showed long grains, even though they carried the *gs3* allele. The main reason was that the dense and erect panicle varieties carried the high-yield-associated *dep1* allele, which showed negative effect on GL (Li et al. 2019; Zhao et al. 2019). Both *GS3* and *DEP1* encodes Gγ protein containing a C-terminal cysteine-rich domain (Botella 2012). The *gs3* allele could enhance the interaction between Gγ subunits *DEP1* and Gβ, resulting in a long and slender grain, while the *dep1* allele could reduce the interaction between *DEP1* and Gβ, resulting in not only dense and erect panicles but also smaller grains (Sun et al. 2018). Recently, a major QTL *qLGY3* has been identified from American *japonica* variety L-204, which could simultaneously improve both the grain yield and GL by combining with *dep1* and *gs3* alleles (Liu et al. 2018). These results indicated that discovering novel genes associated with GL in *japonica* germplasms would be of great importance to improve grain size of erect panicle varieties.

In our previous studies, to improve the GL of erect panicle varieties, the *japonica* variety Shennong9017 with long grains was used as parent to cross with dense and erect panicle variety Liaojing454 to thus develop a *japonica* super rice variety Liaoxing1 (LX1). In addition to dense and erect panicles and more grains per panicle, one of the most significant morphological features of LX1 is their long grains, which were higher than those of other dense and erect panicle varieties. The objective of present study was (1) to detect the QTLs for GL by using a recombinant inbred lines (RILs) population derived from a cross between Gangyuan8 (GY8) and LX1 in four environments, (2) to localize the major-effect QTL *qGL6.1* to a narrow genomic region by using the BC_1_F_2_ population, and (3) to identify the candidate gene of *qGL6.1*. Our results will provide important information to unearth the QTL for rice GL and help to understand how to achieve high yield and long grains in the LX1.

## Results

### Phenotypic Variation of Grain Length in the RIL Population

**Fig. 1:**
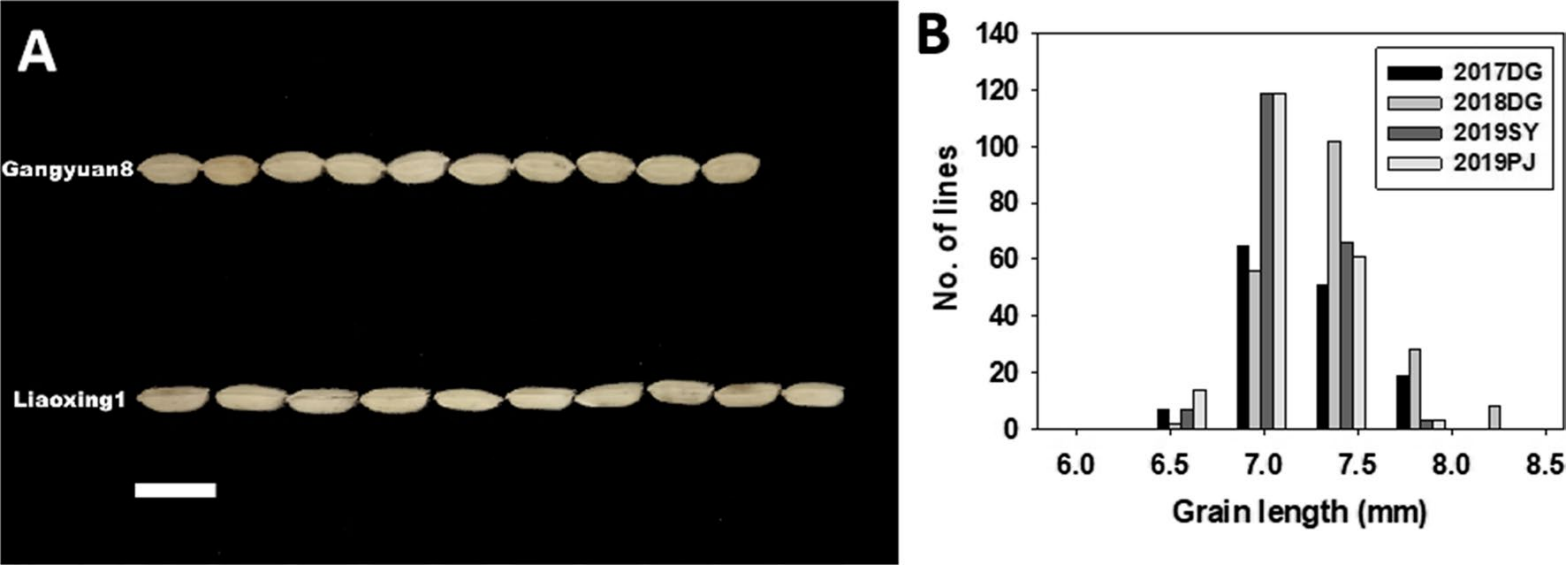
Difference of grain length between Gangyuan8 and Liaoxing1 (**a**) and the frequency distributions of grain length (**b**) in the RIL population in four environments. DG, Donggang; SY, Shenyang; PJ, Panjin

There was significant genetic variation in GL among each RIL line and two parents in all environments. LX1 and GY8 gave averaged GL of 7.6 mm and 6.7 mm, respectively (Fig. 1A). GL of the RILs ranged from 6.55 mm to 8.02 mm in each environment and exhibited approximately normal distributions (Fig. 1B), indicating that they controlled by multiple genes. Pearson’s correlation coefficients among the four environments and BLUP ranged from 0.596 to 0.906 (*P* < 0.01), indicating that the GL was consistent across environments (Additional file 1: Table S1). The ANOVAs showed significant phenotypic variation in GL among genotypes, environments and genotype × environment interactions, and the variation among replications within experiments was no significant. More than 79% heritabilities were estimated for GL in four environments (Additional file 1: Table S2), indicating that the QTL with large effect for GL could be detected in this study.

### QTL Analysis in Individual Environments and Their BLUP Values

The phenotypic values of GL in four environments and its BLUP values were determined using the ICIM-ADD mapping method in BIP module for QTL mapping. A total of 20 QTLs that controlled GL was detected in the individual environment, which included two QTL in 2018DG, four QTLs in 2019DG, four QTLs in 2019PJ, four QTLs in 2019SY and six QTLs in BLUP values, explaining 30.03%, 32.26%, 36.94%, 38.69% and 50.31% of the phenotypic variation, respectively (Table 1). We identified eight single QTL with phenotypic contributions of 10%-20%. Fourteen favorable QTL alleles were from the long grain parent LX1, whereas 6 favorable QTL alleles emerged from GY8.

**Table 1:** QTLs for grain length in rice by single environment analysis

QTL	Environment	Chromosome	Marker interval	ICIM		
				LOD	PVE (%)	Add
*qGL5.1*	2018DG	5	AX-153909583 ~ AX-154483228	5.14	7.18	− 0.09
*qGL5.2*	2017DG	5	AX-95927242 ~ AX-95926505	3.40	8.35	− 0.09
*qGL5.3*	2019SY	5	AX-95952779 ~ AX-154380199	4.55	7.22	− 0.06
*qGL5.3*	BLUP	5	AX-95952779 ~ AX-154380199	6.18	7.63	− 0.07
*qGL5.3*	2019PJ	5	AX-154380199 ~ AX-154326915	3.19	4.87	− 0.05
*qGL6.2*	BLUP	6	AX-116842541 ~ AX-95937303	2.53	7.29	− 0.07
*qGL6.1*	2017DG	6	AX-95928617 ~ AX-95962924	7.85	21.68	− 0.14
*qGL6.1*	2018DG	6	AX-95928617 ~ AX-95962924	9.33	14.14	− 0.12
*qGL6.1*	2019SY	6	AX-95928617 ~ AX-95962924	9.35	15.92	− 0.09
*qGL6.1*	2019PJ	6	AX-95928617 ~ AX-95962924	8.48	13.53	− 0.09
*qGL6.1*	BLUP	6	AX-95928617 ~ AX-95962924	11.76	15.56	− 0.09
*qGL7.2*	2018DG	7	AX-95957416 ~ AX-95957100	3.57	4.97	0.08
*qGL7.2*	BLUP	7	AX-95957416 ~ AX-95957100	3.00	3.53	0.05
*qGL9*	2018DG	9	AX-153942631 ~ AX-115845259	3.29	5.97	0.08
*qGL10*	2019SY	10	AX-154280635 ~ AX-115738695	3.18	5.40	0.07
*qGL10*	2019PJ	10	AX-154280635 ~ AX-115738695	3.92	6.49	0.07
*qGL10*	BLUP	10	AX-154280635 ~ AX-115738695	3.51	4.74	0.06
*qGL11*	2019SY	11	AX-115836788 ~ AX-115753113	6.03	10.15	− 0.08
*qGL11*	2019PJ	11	AX-115836788 ~ AX-115753113	6.52	12.05	− 0.08
*qGL11*	BLUP	11	AX-115836788 ~ AX-115753113	7.67	11.56	− 0.08

QTL *qGL6.1* detected simultaneously by BLUP data and was consistent across four environments, was flanked by AX-95928617 and AX-95962924 on chromosomes 6. Two QTL *qGL5.3* and *qGL11* detected simultaneously by BLUP data and were consistent across two environments, were flanked by AX-95952779 and AX-154326915 on chromosomes 5 and AX-115836788 and AX-115753113 on chromosomes 11, respectively. *qGL6.1*, *qGL5.3* and *qGL11* had positive effect on GL, explaining 13.53–21.68%, 4.87–7.63% and 10.15–12.05% of the total phenotypic variation, respectively, and the positive allele came from the same donor LX1.

### Joint Analysis in Multiple Environments

**Fig. 2:**
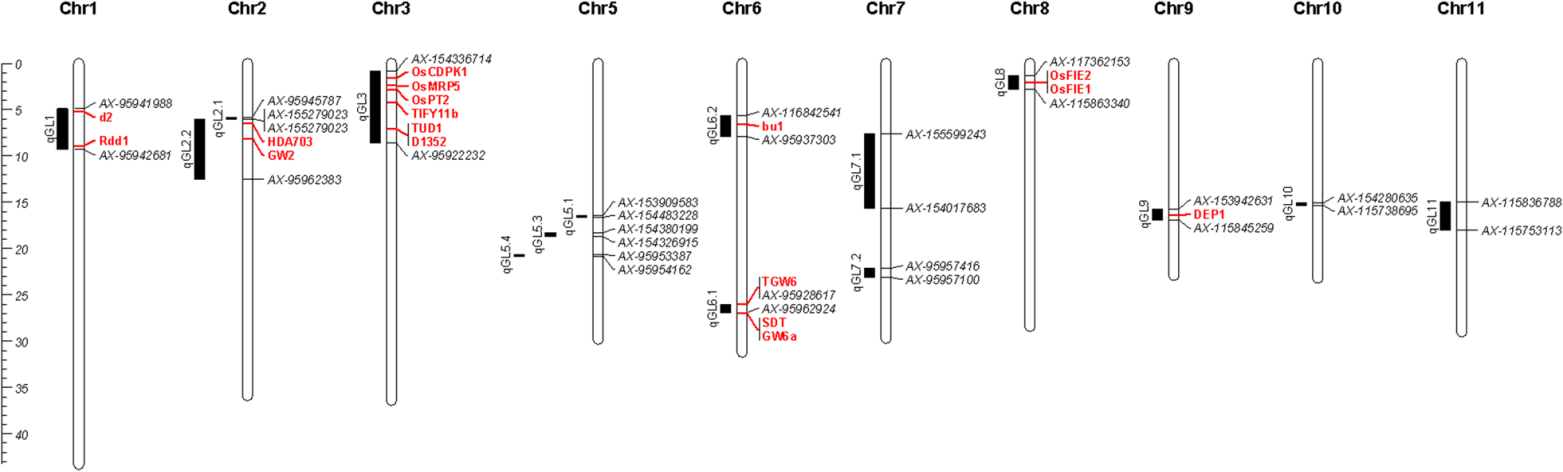
Physical map showing the QTLs identified for grain length in RIL population by individual environmental analyses. Red font, known genes; black font, the markers and QTL detected in this study

A total of 15 QTLs that controlled GL was detected based on joint analysis in multiple environments and showed in the physical map (Fig. 2). Three stable QTL *qGL5.3*, *qGL6.1* and *qGL11* detected in individual environment analyses were all mapped to the same marker intervals to those detected by the joint analysis (Table 2). By using the ICIM-ADD mapping method in the MET module, the *qGL5.3* and *qGL11* showed significant additive effect (LOD(A) > 2.5) as well as QTL × environment interaction effects (LOD(AbyE) > 2.5), but these additive contribution rates were higher than those between the additive effect and environment interaction, indicating that these additive effects were the main contributors to phenotypic variation. The *qGL6.1* had significant additive effect (LOD(A) > 2.5) rather than additive-environment interaction (LOD(AbyE) < 2.5), which were considered to be expressed independently. These three QTL *qGL5.3*, *qGL6.1* and *qGL11* were detected by the joint analysis with phenotypic contribution of 2.86%, 17.51% and 5.45%, respectively.

**Table 2:** Detection of rice grain length QTLs based on joint analysis in multiple environments

QTL	Chromosome	Marker interval	LOD		PVE (%)		Add			
			A	AbyE	A	AbyE	2017DG	2018DG	2019SY	2019PJ
*qGL1*	1	AX-95941988 ~ AX-95942681	3.23	0.26	1.42	0.09	0.00	0.00	0.01	− 0.01
*qGL2.1*	2	AX-95945787 ~ AX-155279023	2.09	0.77	0.94	0.20	0.01	− 0.01	0.01	− 0.01
*qGL2.2*	2	AX-155279023 ~ AX-95962383	2.88	0.34	1.29	0.08	0.00	0.00	0.01	− 0.01
*qGL3*	3	AX-154336714 ~ AX-95922232	2.79	0.09	1.25	0.02	0.00	0.00	0.00	0.01
*qGL5.1*	5	AX-153909583 ~ AX-154483228	2.03	3.02	0.93	2.30	0.03	− 0.06	0.02	0.02
*qGL5.3*	5	AX-154380199 ~ AX-154326915	4.33	3.59	1.96	0.90	0.03	0.02	− 0.02	− 0.03
*qGL5.4*	5	AX-95953387 ~ AX-95954162	2.57	0.12	1.16	0.10	− 0.01	0.00	0.01	0.01
*qGL6.2*	6	AX-116842541 ~ AX-95937303	2.36	0.42	0.95	0.29	0.01	− 0.03	0.01	0.00
*qGL6.1*	6	AX-95928617 ~ AX-95962924	33.91	0.83	17.20	0.31	0.00	− 0.02	0.01	0.01
*qGL7.1*	7	AX-155599243 ~ AX-154017683	1.25	1.73	0.56	0.47	− 0.01	− 0.03	0.02	0.01
*qGL7.2*	7	AX-95957416 ~ AX-95957100	7.41	0.10	3.33	0.47	0.00	0.03	− 0.02	− 0.01
*qGL8*	8	AX-117362153 ~ AX-115863340	4.06	0.26	1.82	0.11	− 0.01	− 0.01	0.00	0.01
*qGL9*	9	AX-153942631 ~ AX-115845259	6.68	0.10	2.89	0.38	0.00	0.02	− 0.01	− 0.01
*qGL10*	10	AX-154280635 ~ AX-115738695	5.16	2.46	2.67	0.42	− 0.03	− 0.01	0.02	0.02
*qGL11*	11	AX-115836788 ~ AX-115753113	10.31	3.51	4.91	0.54	0.02	0.01	− 0.02	− 0.02

The underline represented that these QTLs were also detected in individual environment analyses

### Fine Mapping of *qGL6.1* and Analyzing Candidate Genes

**Fig. 3:**
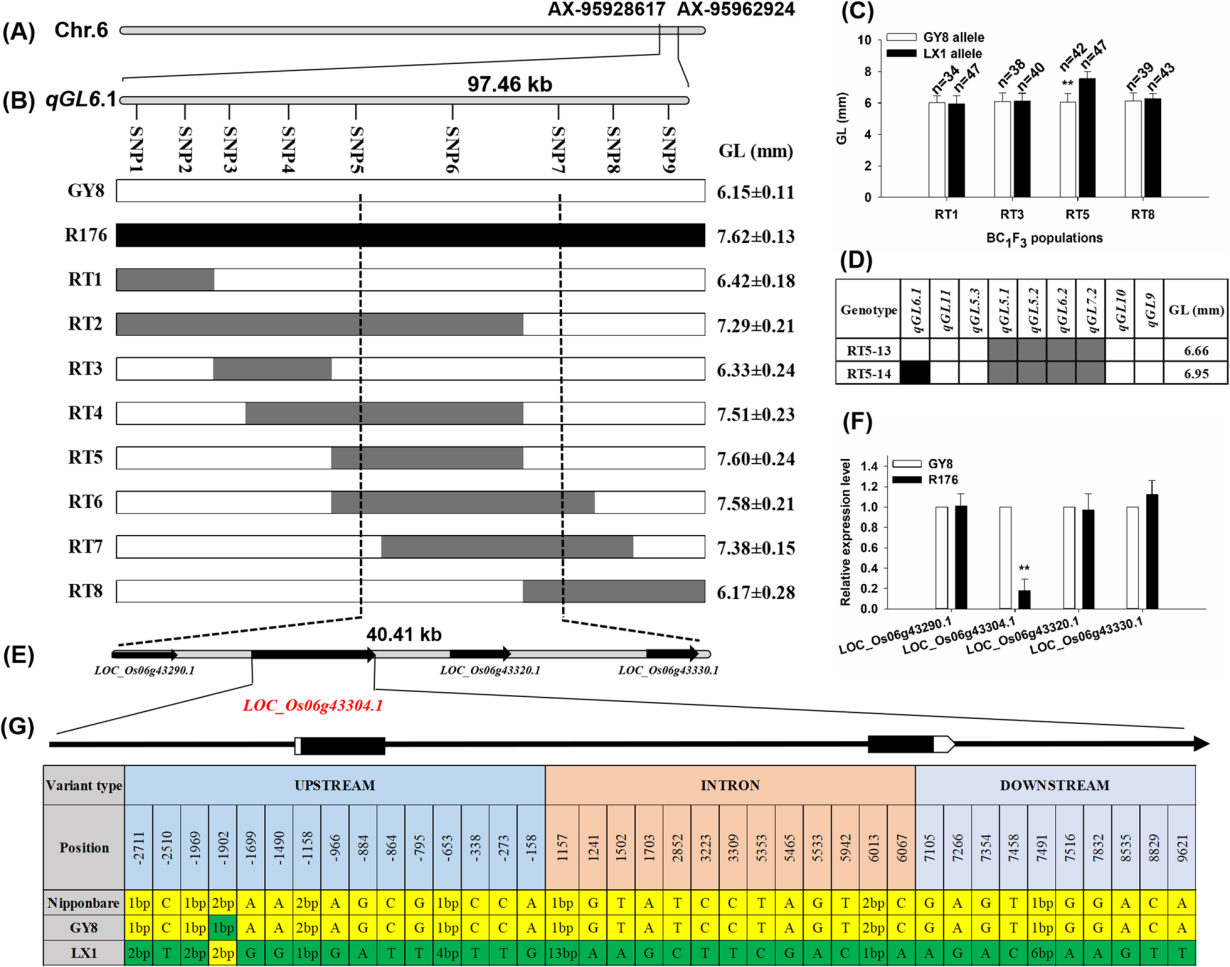
Fine mapping of *qGL6.1* and and analyzing candidate genes. **a** The physical map of *qGL6.1* region on chromosome 6. **b** Fine mapping of *qGL6.1* with eight recombinants. The black bar with markers denotes the recombinant sites. The white, black and gray boxes stand for genotypes of Gangyuan8, R176 and heterozygote, respectively. Vertical dotted lines denote the target region. **c** The progeny test in the BC_1_F_3_ populations derived from the heterozygous recombinants R1, R3, R5 and R8. **d** Two BC_1_F_3_ plants derived from heterozygous recombinants R5 carried different alleles at qGL6.1 locus without the interference of other QTLs. **e** Four candidate genes in the fine mapping region. Arrows indicate direction of gene orientation. **f** The expression level of four candidate genes by qPCR analysis. **g** Variations in the upstream, intron and downstream region of *LOC_Os06g43304.1* among Nipponbare, Gangyuan8 (GY8) and Liaoxing1 (LX1)

By individual environmental analyses and multi-environment joint analysis, the major-effect QTL *qGL6.1* for GL was detected in an approximate 97.46-kb interval on chromosomes 6 (Fig. 3A). To obtain a candidate genomic region for the positioning of *qGL6.1*, the line R176 with long grains (7.62 mm) to backcrossed with recipient parent GY8, as they carried different allele in *qGL6.1* locus but had 68% similar in genetic background by 8 K-SNP chip analysis. A total of 2, 487 BC_1_F_2_ plants were detected by nine KASP markers across the *qGL6.1* target region to identify eight key informative recombinants (Fig. 3B). Among them, the heterozygous recombinants RT2, RT4, RT5, RT6 and RT7 showed longer grains compared with the others. The progeny test was further conducted in the BC_1_F_3_ populations derived from the heterozygous recombinants RT1, RT3, RT5 and RT8. Only in the population derived from RT5, the homozygotes with LX1 allele had longer grains than those with GY8 allele (Fig. 3C). The difference of GL between these progenies with LX1 and GY8 allele at *qGL6.1* locus was more than 1.4 mm, because the phenotypic variation was affected by other QTLs, such as *qGL11*, *qGL5.3*, *qGL5.1*, *qGL5.2*, *qGL6.2*, *qGL7.2* and *qGL10* (Additional file 2: Fig. S1). To eliminate the interference of these QTLs, we found two type of plants (RT5-13 and RT5-14) carried same alleles at *qGL11*, *qGL5.3*, *qGL5.1*, *qGL5.2*, *qGL6.2*, *qGL7.2* and *qGL10* rather than *qGL6.1* locus. The RT5-14 with LX1 allele at *qGL6.1* locus showed an increase of 0.29 mm in GL than RT5-13 with GY8 allele (Fig. 3D). Finally, the *qGL6.1* locus was narrowed down to a 40.41-kb genomic interval with flanking markers SNP5 (Chr6: 26,011,771) and SNP7 (Chr6: 26,052,181) (Fig. 3E). According to the Rice Annotation Project annotation (http://rapdb.dna.affrc.go.jp/), this region contains four annotated genes, *LOC_Os06g43290.1*, *LOC_Os06g43304.1*, *LOC_Os06g43320.1* and *LOC_Os06g43330.1.* The *LOC_Os06g43290.1* and *LOC_Os06g43330.1* encodes putative Ty1-copia subclass retrotransposon protein, and *LOC_Os06g43304.1* and *LOC_Os06g43320.1* encodes cytochrome P450, respectively.

By qPCR analysis in the inflorescence meristem, only the *LOC_Os06g43304.1 differentially expressed* between GY8 and R176 among these four annotated genes (Fig. 3F). The expression of *LOC_Os06g43304.1* was down-regulated in R176 as compared to GY8. We also found 10 SNP and 5 InDel in upstream region, 11 SNP and 2 InDel in intron region, as well as 9 SNP and 1 InDel in downstream region, respectively, in the DNA sequence of *LOC_Os06g43304.1* between GY8 and LX1 (Fig. 3G). Through gene prediction and sequencing, the *LOC_Os06g43304.1* encoding cytochrome P450 in rice, was identified as a candidate gene of *qGL6.1*.

## Discussion

In this study, we sought to identify novel genes that control GL in rice by using the RIL population in four environments to dissect the genetic basis of the long grains *japonica* variety. As we known, the QTL detected in multiple environments could be more stable than QTL with high effect values detected in a single environment and have a more useful value for MAS breeding (Fulton et al. 1997). We simultaneously detected three QTL *qGL5.3*, *qGL6.1* and *qGL11* associated with GL regulation in the *japonica* variety LX1 by individual environmental analyses and multi-environment joint analysis, while their additive effects had higher phenotypic contributions than QTL × environment interactive effects. Among them, *qGL6.1* were identified by BLUP data and were consistent across four environments, so it can be considered as stable QTL for GL. Importantly, the *qGL6.1* that explained 13.53–21.68% and 17.51% of the phenotypic variation by individual environment analyses and joint analysis, respectively, could be identified as the major-effect QTL for GL.

By using the RIL population, the *qGL6.1* was detected in an approximate 97.46 kb interval on chromosome 6 between AX-95928617 and AX-95962924. The *qGL6.1* was compared to QTL that had been previously reported for the same or related traits by physical positions of the respective interval markers. The position of *qGL6.1* differed slightly from those of known QTL cloned as single genes related to GL on chromosome 6, like *TGW6* (Ishimaru et al. 2013), *GL6* (Wang et al. 2019), *SDT* (Zhao et al. 2015), *qGL-6* (Zhang et al. 2020) and *GW6a* (Song et al. 2015; Gao et al. 2021). The DNA sequences analysis showed none polymorphism in the promoter or coding region of *GW6a* (*LOC_Os06g44100*)*, SDT* (*LOC_Os06g44034*) and *qGL-6* (*LOC_Os06g48950*) between the GY8 and LX1 (Additional file 3: Fig. S2A–C). Although six nucleotide substitutions were found in coding regions of *TWG6* (*LOC_Os06g41850*) between GY8 and LX1, they not expected to affect the protein (Additional file 3: Fig. S2D). In previous study, the premature stop codon by a nucleotide substitutions was found in *GL6* (*LOC_Os06g45540*), which cause reduction or loss of function of GL6, thus affecting rice grain length (Wang et al. 2019). However, the long grains *japonica* variety LX1 only showed a loss of Arg by deletion of nucleotide GAC in the coding region of *GL6* (Additional file 3: Fig.S2E)*.* Inportantly, there were non-significant different in the expression levels of *SDT*, *qGL-6*, *GW6a*, *TGW6* and *GL6* between GY8 and LX1 (Additional file 4: Fig. S3), suggesting that these genes could not explain the variation of GL in this study. Interestingly, the interval of *qGL6.1* was within *qGN6* and *qBNS6.2*, controlling grain number per panicle and number of secondary branches, respectively, which were co-localized in a 0.94 Mb interval on chromosome 6 between AX-95955496 and AX-115755704 in our previous study (Zhao et al. 2020). However, it was unknown whether a cluster of QTLs or a true pleiotropy is the cause of multiple phenotypic variations. We suggested that *qGL6.1* as a novel QTL could be considered as important candidate loci for fine-mapping to identify the molecular regulatory mechanism for GL as well as other panicle components.

For fine mapping of *qGL6.1*, in this study, the line R176 was used as parent to develop the BC_1_F_2_ population, which had 68% similar in genetic background to GY8. Moreover, KASP as the uniplex SNP genotyping platform offers cost-effective and scalable flexibility in QTL fine mapping that require small to moderate numbers of markers (Semagn et al. 2014). By nine KASP markers developed in the target region, eight key informative recombinants were identified in the BC_1_F_2_ population. Although these recombinants shared similar genetic backgrounds with heterozygous rather than homozygous genotypes at the QTL region, they also showed significant difference in GL. We further found significant difference in GL between the homozygotes with LX1 and GY8 allele in a BC_1_F_3_ population. However, the difference of GL was more than 1.4 mm, which almost equals to the range of phenotypic variation in the RILs (Fig. 1B), indicated that the phenotypic difference includes effect of other QTLs. By eliminating the background noise, we found the GL was increased of approximately 0.29 mm by LX1 allele at *qGL6.1* (Additional file 2: Fig. S1), which was consistent with the results of QTLs analysis in single environment (Table 1). Thus, the *qGL6.1* locus was narrowed down to an interval between KASP markers SNP5 (Chr6: 26,011,771) and SNP7 (Chr6: 26,052,181), covering a 40.41-kb region in the Nipponbare genome.

In *qGL6.1* interval, only the *LOC_Os06g43304.1* encoding a cytochrome P450 (CYP71D55) was significantly differentially expressed in the inflorescence meristem at the stage of primary and secondary rachis branch formation between GY8 and R176, which was most likely caused by the variations in its upstream and downstream region. As we known, the cytochrome P450 monooxygenases catalyze numerous monooxygenation/hydroxylation reactions, play an important role in different biochemical pathways, plant metabolism, cell proliferation, and expansion (Nelson et al. 2008; Schuler and Werck-Reichhart 2003). The CYP71D55 as the member of cytochrome P450 family was found to catalyze the successive regio-selective oxidation at the carbon atom C-2 position of premnaspirodiene to yield solavetivone (Banerjee and Hamberger 2018). Although the function of CYP71D55 in rice was unknown, it was reported that other homologous members regulated the grain size in *Arabidopsis*, wheat and rice. In *Arabidopsis*, several cytochrome P450s, including CYP78A5, CYP78A6, CYP78A7and CYP78A9, have been characterized as the positive regulators of seed size, which acts through a non-cell-autonomous signal to promotes organ growth (Adamski et al. 2009; Stransfeld et al. 2010; Sotelo-Silveira et al. 2013). In rice, the global identification, structural analysis and expression characterization of cytochrome P450 superfamily have been systematically studied, which provided a clue to understanding biological function of cytochrome P450 in development regulation and drought stress response (Wei and Chen 2018). The *d11* gene, encodes CYP724B1, was implicated in brassinosteroid biosynthesis via the characterization of a rice dwarf mutant, *dwarf11*, with reduced GL in rice (Tanabe et al. 2005). The *BG2* and *GL3.2* encodes CYP78A13 are also responsible for grain size and variations in the exon regions of these genes determined the difference in grain yield (Xu et al. 2015). By referring the function of its homologous genes, therefore, we suggested that the *LOC_Os06g43304.1* encoding CYP71D55 in rice might be the candidate gene for GL in the QTL, *qGL6.1*.

## Conclusions

In this study, using the RIL population, three QTL *qGL5.3*, *qGL6.1* and *qGL11* were detected to control GL by individual environmental analyses and multi-environment joint analysis. The positive alleles of these QTLs came from the long grain parent LX1. With the BC1F2 population, a major-effect and stable QTL *qGL6.1* was fine mapped within 40.41 kb physical interval on chromosome 6. One candidate gene *LOC_Os06g43304.1* encoding cytochrome P450 (CYP71D55) was finally selected based on the difference in the transcriptional expression. The cloning and genetic mechanism study of the *qGL6.1* will facilitate improving appearance quality in *japonica*, especially in erect panicle varieties.

## Materials and Methods

### Plant Materials

The parental lines in this study were the long grains *japonica* variety LX1 and short grains *japonica* variety GY8. The mapping population consisted of 197 F_6:9_ and F_6:10_ RILs developed from a cross between GY8 × LX1.

### Field Trials and Trait Measurement

All 197 RILs and parents were grown at Donggang (39°87′ N, 124°15′ E), Shenyang (41°48′ N, 123°25′ E) and Panjin (41°12′ N, 122°07′ E) in Liaoning Province in 2018 and 2019 cropping seasons (hereafter referred as 2018DG and 2019DG, 2019SY, and 2019PJ) for evaluation of GL. Each RIL and parents were sown in a seedling nursery on April and with one seedling being transplanted per hill on May 24 ~ 30. A completely random block design was repeated three times in each environment. Each plot area was 2.5 m^2^, and the seedlings were transplanted at a spacing of 30 cm between rows and 13 cm between plants. Protection lines were set up everywhere, and field management followed local standards of production for rice.

At maturity, the panicles of 10 plants of each line, and their parents were harvested from each plot. The distribution of primary branch number per panicle was firstly measured for each plot. Ten panicles with the largest distribution proportion of primary branch number in each plot were selected to collect the fully filled grains. After the grains were dried for 72 h at 50℃, the lengths of twenty randomly chosen grains from each plot were estimated as the lengthwise distance between opposite tips using a vernier caliper, and the values were averaged and used as the measurements for the line. In 2018DG, the GL of only 142 RILs were measured because of serious lodging of some lines, while that of all RILs were measured in the other three environments.

### Phenotypic Data Analyses

The phenotypic values of RILs in each environment were analyzed. SPSS 19.0 was used for correlation analysis, and AOV function in QTL IciMapping (version 4.2) was used for variance analysis, with default parameters. The information in the ANOVA table was used to calculate broad sense heritability (H2) of each trait: H^2^ = σg^2^/(σ_g_^2^ + σ_ge_^2^ /e + σ_ε_^2^/re), where σ_g_^2^ is (MS_f_ − MS_fe_)/re, σg_e_^2^ is (MS_fe_ − MS_e_)/r and σ_ε_^2^ is MS_e_; σ_g_^2^ = genetic variance, σ_ge_^2^ = genotype × environment interaction variance, σ_ε_^2^ = error variance, MS_f_ = mean square of genotypes, MS_fe_ = mean square of genotype × environment interaction, MS_e_ = mean square of error, r = number of replications, and e = number of environments.

The best linear unbiased prediction (BLUP) of each parameter is calculated in the mixed linear model by using the lmer function in package lme4 of R software (Bates et al. 2012) (http://www.R-project.org/). Genotype, environment, genotype × environment interaction, and nested repeats in the environment are all considered random factors.

### Genotyping

Genomic DNA was extracted from the fresh leaves of each line using the cetyl trimethyl ammonium bromide method (Murray and Thompson 1980). The whole genome resequencing of parents GY8 and LX1 was conducted by Biomarker Technologies. Each RIL (n = 197 genotypes) along with their parents were genotyped using the 8 K-SNP chip by Beijing Golden Marker Biotechnology Co., Ltd (Beijing, China). The SNP markers were screened as follows: ambiguous SNP calling, simplex and poor quality SNP loci with > 10% missing values, or minor allele frequencies < 0.05 were removed in further analysis.

### Linkage Mapping and QTL Analysis

After removing the redundant markers, QTL IciMapping 4.2 software was used to generate an input file for genetic map construction (Meng et al. 2015). In this genetic linkage maps, a total of 559 SNPs covering the 12 chromosomes were polymorphic between Gangyuan8 and Liaoxing1 (Zhao et al. 2020). In an individual environmental analysis, the inclusive composite interval mapping (ICIM) method was conducted using the BIP function to detect the additive QTL for phenotypic value and BLUP value of panicle traits (Li et al. 2007). A 1000-permutation test was conducted at a 95% confidence level, and an LOD threshold of 2.5 was used. In addition, the multi-environmental joint analysis utilized the ICIM method in the MET functional module to detect the QTL additive and QTL × environment interaction effects (Li et al. 2015).

QTL were named using the abbreviated English name of trait with “q” before it, followed by the chromosome number or chromosome number plus an ordered number designating one of multiple QTL in a single chromosome. The QTL detected in this study were compared to the Q-TARO database (Yonemaru et al. 2010).

### Fine-Mapping by Using BC_1_F_2_ Population

After phenotypic and genotypic data analysis, one line with long grains, carrying the LX1 allele in major-effect QTL for GL but having similar genetic background to GY8, was selected in RIL population. For QTL fine-mapping, this line was backcrossed with GY8 to generate the BC_1_F_2_ population. A total of 2, 487 BC_1_F_2_ plants were grown at SY in 2021 cropping seasons for evaluation of GL. DNA was isolated from the two parental lines and the BC_1_F_2_ population. According to the SNPs in the QTL region obtained from deep re-sequencing of two parental lines, the informative KASP markers (Additional file 1: Table S3) were developed to genotype each plant of the BC_1_F_2_ population. A total of 753 BC_1_F_3_ plants derived from the heterozygous recombinants were further identified at Hainan Province, China, in the winter of 2021. To eliminate the interference of other QTLs, the informative KASP markers (Additional file 1: Table S4) were also developed to genotype the plants of the BC_1_F_3_ population. KASP assays were conducted in a 1536-well plate format using the protocol of LGC Genomics (LGC, Middlesex, UK). The Synergy H1 fullfunction microplate reader (FLUO star Omega, BMG Labtech, Germany) was used to read the fluorescence signal. Then the linkage relationship between markers and the *qGL6.1* locus was analysed for fine-mapping.

### RNA Isolation and qPCR Analysis

To identify the expression of candidate genes, at the stage of primary and secondary rachis branch formation, the inflorescence meristem of GY8, LX1 and R176 were sampled for RNA extraction and qPCR analysis. Total RNA was extracted with TriZol reagent (Invitrogen, Germany) according to the manufacturer’s instructions. The first strand of cDNAs were synthesized from DNaseI-treated total RNA using a Primer Script RT reagent Kit with gDNA Eraser (Takara, Japan). Reverse-transcribed RNA was used as PCR template for gene-specific primers (Additional file 1: Table S5). Each reaction contained 3.0 μl of first-strand cDNAs, 2 μl of 200 μM gene-specific primers, and 12.5 μl of 2 × SYBR Green Master Mix reagent (Applied Biosystems) in a final volume of 25 μl. The qPCR analysis was performed in a real-time PCR system (BIO-RAD). The qRT-PCR analysis was performed for each cDNA sample with four replications. Relative expression levels were calculated by 2^−△△CT^ (Livak and Schmittgen 2001).

### PCR and Sequencing

To identify candidate gene, the upstream region (2000 bp), exon and intron region, as well as downstream region (1000 bp) of related genes were amplified from genomic DNA using KOD plus Neo FX (Toyobo, Japan). The primer used to sequence these locus was listed in Additional file 1: Table S6. The sequencing of the PCR production was performed at Quintara Biosciences (Wuhan, China). The genomic sequences were assembled by ContigExpress and aligned by DNAStar.

## Supplementary Information

**Additional file 1**. **Table S1** Correlation of grain length in different environments. **Table S2** Grain length of rice recombinant inbred lines in different environments. **Table S3** The Kompetitive Allele Specific PCR markers for fine mapping of *qGL6.1*. **Table S4** The Kompetitive Allele Specific PCR markers for seven QTLs in this study. **Table S5** The gene-specific primers for qPCR. **Table S6** The gene-specific primers for DNA sequencing.

**Additional file 2**. **Fig. S1** The genotypic data at *qGL11*, *qGL5.3*, *qGL5.1*, *qGL5.2*, *qGL6.2*, *qGL7.2* and *qGL10* locus of some progenies derived from the heterozygous recombinants R5.

**Additional file 3**. **Fig. S2** Variations in the upstream, intron and downstream region of *GW6a* (A), *SDT* (B), *qGL-6* (C), *TGW6* (D) and *GL6* (E) between Gangyuan8 (GY8) and Liaoxing1 (LX1).

**Additional file 4**. **Fig. S3** The expression level of *GW6a*, *SDT*, *qGL-6*, *TGW6* and *GL6* by qPCR analysis.

## Data Availability

The datasets supporting the conclusions of this article are included within the article.

## Abbreviations

QTL: Quantitative trait loci
GL: Grain length
RILs: Recombined inbred lines
KASP: Kompetitive allele specific PCR
DG: Donggang
SY: Shenyang
PJ: Panjin
