# Supplementary material for: QTL Detection for Rice Grain Length and Fine Mapping of a Novel Locus qGL6.1
Source: Rice (N Y). 2022 Nov 28;15:60. doi: 10.1186/s12284-022-00606-z (PMC9705657; doi:10.1186/s12284-022-00606-z)
Supplement: Supplementary file 1 — Additional file 1. Table S1 Correlation of grain length in different environments. Table S2 Grain length of rice recombinant inbred lines in different environments. Table S3 The Kompetitive Allele Specific PCR markers for fine mapping of qGL6.1. Table S4 The Kompetitive Allele Specific PCR markers for seven QTLs in this study. Table S5 The gene-specific primers for qPCR. Table S6 The gene-specific primers for DNA sequencing. [file 12284_2022_606_MOESM1_ESM.docx]

**Table S1 Correlation of grain length in different environments**

| Environment | 2018DG | 2019DG | 2019SY | 2019PJ | BLUP |
| --- | --- | --- | --- | --- | --- |
| 2018DG | 1 |  |  |  |  |
| 2019DG | 0.697** | 1 |  |  |  |
| 2019SY | 0.646** | 0.662** | 1 |  |  |
| 2019PJ | 0.596** | 0.664** | 0.679** | 1 |  |
| BLUP | 0.849** | 0.847** | 0.850** | 0.906** | 1 |

**Table S2 Grain length of rice recombinant inbred lines in different environments**

| Environment | Gangyuan8 | Liaoxing1 | Mean | Range | Skewness | Kurtosis | σ_g_^2^ | h _b_^2^ |
| --- | --- | --- | --- | --- | --- | --- | --- | --- |
| 2018DG | 6.71 | 7.31 | 7.24 | 6.63~8.02 | 0.28 | -0.44 | 0.11 | 0.92 |
| 2019DG | 6.82 | 7.86 | 7.38 | 6.77~8.31 | 0.67 | 0.65 | 0.08 | 0.89 |
| 2019SY | 6.65 | 7.58 | 7.09 | 6.55~7.79 | 0.18 | 0.02 | 0.05 | 0.88 |
| 2019PJ | 6.81 | 7.61 | 7.12 | 6.60~7.87 | 0.38 | 0.09 | 0.04 | 0.79 |
| BLUP | - | - | 7.18 | 6.71~7.89 | 0.50 | 0.01 | - | - |

**Table S3** The Kompetitive Allele Specific PCR markers for fine mapping of *qGL6.1*.

| Number | Position | FAM primer(5'-3') | HEX primer(5'-3') | Common primer(5'-3') |
| --- | --- | --- | --- | --- |
| SNP1 | Chr6:25974163 | GAAGGTGACCAAGTTCATGCTTGAGTGTATGTGTTCTGTA | GAAGGTCGGAGTCAACGGATTTGAGTGTATGTGTTCTGTG | TGGTTATAGGTAGATCTCTATTT |
| SNP2 | Chr6:25982137 | GAAGGTGACCAAGTTCATTTGACCGCTTATTTTTCTG | GAAGGTCGGAGTCAACGGATTTGACCGCTTATTTTTCTA | ATAAATTTTAAAAATAGATTTGTT |
| SNP3 | Chr6:25989874 | GAAGGTGACCAAGTTCATGCTTTGTCCGGTGTCTCTTTGCCT | GAAGGTCGGAGTCAACGGATTTTGTCCGGTGTCTCTTTGCCC | CTGATGAATTGTTGCGCGCGACA |
| SNP4 | Chr6:26001319 | GAAGGTGACCAAGTTCATTCAGGGTGGTTACTTCAC | GAAGGTCGGAGTCAACGGATTTCAGGGTGGTTACTTCAG | AAGATTAGTTGCTGAGCCCTTTCT |
| SNP5 | Chr6:26011771 | GAAGGTGACCAAGTTCATTGGCAAGTGAGATGTACAGC | GAAGGTCGGAGTCAACGGATTTGGCAAGTGAGATGTACAGT | TGGCTCTCCAGGCCTTTGCATAGCTT |
| SNP6 | Chr6:26031888 | GAAGGTGACCAAGTTCATTAATTTCGATCACAAA | GAAGGTCGGAGTCAACGGATTTAATTTCGATCACAAT | TTTGCATAGCTTATGGTGTACTTGAA |
| SNP7 | Chr6:26052181 | GAAGGTGACCAAGTTCATAAATAGAAAAAACCACCCCT | GAAGGTCGGAGTCAACGGATTAAATAGAAAAAACCACCCCG | TATATAAATAGGAAGGGGTAGAGTGGA |
| SNP8 | Chr6:26059698 | GAAGGTGACCAAGTTCATGCTACTAGGCCGGTGGCCCAAC | GAAGGTCGGAGTCAACGGATTACTAGGCCGGTGGCCCAAT | AGAGTAACTATGTATGGGCACAGTA |
| SNP9 | Chr6:26068862 | GAAGGTGACCAAGTTCATGCTATTTATTGCGGTTTGGACGA | GAAGGTCGGAGTCAACGGATTATTTATTGCGGTTTGGACGG | TCGCCACCTAACCCACGATCTGA |

**Table S4** The Kompetitive Allele Specific PCR markers for seven QTLs in this study.

| QTL | Position | FAM primer(5'-3') | HEX primer(5'-3') | Common primer(5'-3') |
| --- | --- | --- | --- | --- |
| *qGL5.1* | Chr5:16444726 | GAAGGTGACCAAGTTCATGCTCTGTAACCACTATAGCAATCAGACAATAGT | GAAGGTCGGAGTCAACGGATTCTGTAACCACTATAGCAATCAGACAATAGC | AGAAGGTGCAACTGATCAGGA |
| *qGL5.1* | Chr5:16600568 | GAAGGTGACCAAGTTCATGCTACGTACTGCACCAAAAAGATAACAGT | GAAGGTCGGAGTCAACGGATTACGTACTGCACCAAAAAGATAACAGC | CAATGGATGCATGCTTAATTATGACAA |
| *qGL5.2* | Chr5:16664316 | GAAGGTGACCAAGTTCATGCTGATCATCCAAAGTTACCATGTCCAACATT | GAAGGTCGGAGTCAACGGATTGATCATCCAAAGTTACCATGTCCAACATC | GCCTGACAGCAACAACAGTG |
| *qGL5.2* | Chr5:16700399 | GAAGGTGACCAAGTTCATGCTGAAATTGAGCAGCTCGGATGCT | GAAGGTCGGAGTCAACGGATTGAAATTGAGCAGCTCGGATGCC | AGTAGAGCTGTGGCAAGCAA |
| *qGL5.3* | Chr5:18075626 | GAAGGTGACCAAGTTCATGCTTCTCCGACATCATCACCCGG | GAAGGTCGGAGTCAACGGATTTCTCCGACATCATCACCCGT | CACAAAGGGTTCCATCGCAG |
| *qGL5.3* | Chr5:18271181 | GAAGGTGACCAAGTTCATGCTATGTGATCTTGACATTTTTAAACTTAGA | GAAGGTCGGAGTCAACGGATTTGTGATCTTGACATTTTTAAACTTAGC | TTTTGAATTTTCTTTGCTCTAAACTC |
| *qGL6.2* | Chr6:5603220 | GAAGGTGACCAAGTTCATGCTGGGTCTTATGGGTATATACGGACGC | GAAGGTCGGAGTCAACGGATTGGGTCTTATGGGTATATACGGACGA | TGCCATCACTGTACCTTGCA |
| *qGL6.2* | Chr6:7921570 | GAAGGTGACCAAGTTCATGCTAAAGGATGGGAAGCAGCAACAG | GAAGGTCGGAGTCAACGGATTAAAGGATGGGAAGCAGCAACAA | CCATCCCACACCGAAGTCAA |
| *qGL7.2* | Chr7:22118606 | GAAGGTGACCAAGTTCATGCTAGTATCTGATGAGAATGTTATTATGGACAGATT | GAAGGTCGGAGTCAACGGATTAGTATCTGATGAGAATGTTATTATGGACAGATC | GGCAACAGAAGCAACAGTGT |
| *qGL7.2* | Chr7:23147515 | GAAGGTGACCAAGTTCATGCTCCGGAAGCGTTGTACAAGGTT | GAAGGTCGGAGTCAACGGATTCCGGAAGCGTTGTACAAGGTC | AATGGGTCCTTGAGCAGCTC |
| *qGL10* | Chr10:1039012 | GAAGGTGACCAAGTTCATGCTTCTCAAGGATGATTGTTTCAAGATCCG | GAAGGTCGGAGTCAACGGATTTCTCAAGGATGATTGTTTCAAGATCCA | TCTCCTCCTCCGTGTGGAAA |
| *qGL10* | Chr10:15305723 | GAAGGTGACCAAGTTCATGCTGCTGAATCTCTTTTTGCGGTCATATTTACT | GAAGGTCGGAGTCAACGGATTGCTGAATCTCTTTTTGCGGTCATATTTACC | CATGCAGATCGTCCAAAGCTGT |
| *qGL11* | Chr11:14971311 | GAAGGTGACCAAGTTCATGCTCCAGAGTACATTACACATTCCAAGCTT | GAAGGTCGGAGTCAACGGATTCCAGAGTACATTACACATTCCAAGCTC | CTTCCTGACAGGAGCTGCAG |
| *qGL11* | Chr11:17953878 | GAAGGTGACCAAGTTCATGCTGCGAAGTTGCGATACATGCACC | GAAGGTCGGAGTCAACGGATTGCGAAGTTGCGATACATGCACT | TACTTTGGCCCCTGTCAGGT |

**Table S5** The gene-specific primers for qPCR.

| Gene accession | Forward primers (5'-3') | Reverse primers (5'-3') |
| --- | --- | --- |
| *LOC_Os06g44034* | TTGATTCGATTTCATCTC | CTGCTGCTGATGGAGTGA |
| *LOC_Os06g45540* | CCTCGGAAATGGAGAATC | TCATTTGCGAATACAGCC |
| *LOC_Os06g48950* | CGACCACCAGGTGACCACA | CACTGAACAACAGGAGGAAGGG |
| *LOC_Os06g44100* | CGTCCTCGTCGCAGCAGAA | AGACGCTGAGCACGGCGAACGA |
| *LOC_Os06g41850* | CACAGCCACAACGAGAATG | CATCGCCGACGCCTACAT |
| *LOC_Os06g43290.1* | AAAGTCCAGAGGCAGGTATAAGTCA | CCAATCCCTATTCGGGCACA |
| *LOC_Os06g43304.1* | AGGCGGCGCAGGAGGTGATGAGGA | CATGGAACGGTCGAACGCACGGATGA |
| *LOC_Os06g43320.1* | cgccagcctgctcttccact | gtcatcagggagacgtttcgg |
| *LOC_Os06g43330.1* | gagaaggtcgccaaggag | ttgtggaggcgaggtcggaagc |
| *ACTIN1* | CATCGCCGAGTACTTCTAC | ATCCAAATGTTCCAGAGGCG |

**Table S6** The gene-specific primers for DNA sequencing.

| Gene | Forward primers (5'-3') | Reverse primers (5'-3') |
| --- | --- | --- |
| TGW6 | AACATGTGGAGTGTCTGCAC | CTTTTCTTAGCATACATAAC |
|  | TAGTGTCAACGACTGTACCA | ATCCAATGCCTCATCAACTT |
|  | TGGACATTGATCAGGTTACC | GACAAACAGATCGAGTCACC |
| GL6 | CAGCAGTACGCAGCAGTACG | ACTCGTGAGAGAATATAAAT |
|  | GAGTATCCCAAAACTACAGG | ATGCAAAAGCTAAAATCCTA |
|  | CACCCTCTCCTCCAGGTAAT | ATCAAGATCGGCATGCTTTG |
|  | TCACCTCTGATGAAAAAAGG | TCAAGCAGATGTTGCCAGAG |
|  | ATACTGTGCATTGAAGTGCT | ATCTCGTCAAAACACTGCTT |
|  | TGTCACTGAACAACAGGAGG | CGACCACCTTGCTTTTGGTT |
| SDT | TATGTGGCAATTTGCGTAGT | TTTAAGTAATTATTATATCGG |
|  | ATTCATCTCGCAGTTTTCAG | AGAGATGAAATCGAATCAAG |
|  | TGTCAGCATCTCTCATCACT | CAAACATTTCAAAACTTCAA |
|  | TGACCCATTTGCTGCTGTTC | CTAAACGTCAAATATTTATG |
|  | GTGTGTAGAGTTACATTATT | TAGAAAGACAACTATTATGA |
|  | CCTTAGCAAGCATGAGTAAA | ATGAATAACAACAAAGACAC |
|  | ACGGTCTATCTACAGTGGAT | GTCTGTCCTAGCTTTGCATT |
|  | AGATTTGGACATTTTTTGCC | GGATTTTACAGAATTTTCAC |
| qGL-6 | TTATCAAGGGCCACACTCTT | ATTGGTTCTCCTCTCAAACA |
|  | ACTGTTATATCTCCGAATTA | ATCCCGAACCGCCATTGGAA |
|  | ATTGATTCCTGCTCCGACGA | AAGTACAATGAACAAACAGA |
|  | TTGGTGTTCCATTTTGTTCA | ATACACAATCAATTGAAACA |
|  | ATGTTCCAAGCTACCCAAAT | CGAAAGCGCATCCCTAAAGA |
|  | GTGCTAACCGACAGCCAACT | ATCTGTTCCTGAGGTTTGGT |
|  | TCAGACTCAGAACACCATAA | TGTATTAGCAGCATGAATTT |
|  | TGCTCCTATGTCAGTTCCAA | AATGCGGCCATCTATCCATT |
|  | TATGGATGCATTTGCAGTTG | TTGAGATTTGAAGGACTACA |
|  | GCCAACATGAGTGCTAATTT | TGAGATATCATTAACTGTGA |
| GW6a | AAGTTTGATGTGATCATGTG | AGAGGTAGGGTTAAGAAGAT |
|  | AAGCTCGGCAGCGCGAGCT | CTCTCAACACTCAAGGCTGT |
|  | GCCATTGCTACTTGCTTGCA | AATGAATGGTCAAACAGTGC |
|  | GCTTCAAGACAAATACATTA | GGATCTTGGCCTTGACATTG |
|  | AATATGTCAGACTTGGTTGC | TCAATTTTACGGTTTCACTT |
|  | TTCACCATGAGCCAAATCGC | TGACAGCAGCAGAAATAGAA |
|  | TGATAAAACTGCCAAGGATT | TACTTGAAAGATTGAAACTG |
|  | TACTGCCTAGATTCGTTCGG | ATCCATGAACCTAGGCGAAA |
|  | AGTATCCGTTAATCCGCTCT | TGGCAACAATTACATCTGCT |
|  | GATAAGGATAGGTAGATTTC | ACACAAGCTCACCCTCGGCA |
|  | TTCTTGCGGGCGACGTTGTG | CTCTAATTAAGCCGATCAGA |
|  | ACACCATCAGCCTTGCATTG | ACACTAACTGGTTGAGGAAA |
|  | AACACCTTCATCATCGTCGT | TCACACTACATTGAAAGGAG |
| cytochrome P450 | GCTCAAGCAAGCATGGCGCA | CTGAAAAGAAATGTTGGCAA |
|  | GTTGAAGGTGTGTCCGAAAT | CAGATTATAGATCGGATAAG |
|  | TACTGTCTGGTGCGAGTCAT | GAGATCGTGATCGATGTATT |
|  | GATATGGTATACCTTCATGT | TGAAACATCCGAACAACACT |
|  | CATTTATATTCATTTCATCT | AAGAGATTGGGGTCATTGAT |
|  | TTGCATGTCACTCAAATGGT | ACACCCGTAGTCGGACAAAA |
