# Supplementary figures and images for: QTL Detection for Rice Grain Length and Fine Mapping of a Novel Locus qGL6.1
Source: Rice (N Y). 2022 Nov 28;15:60. doi: 10.1186/s12284-022-00606-z (PMC9705657; doi:10.1186/s12284-022-00606-z)

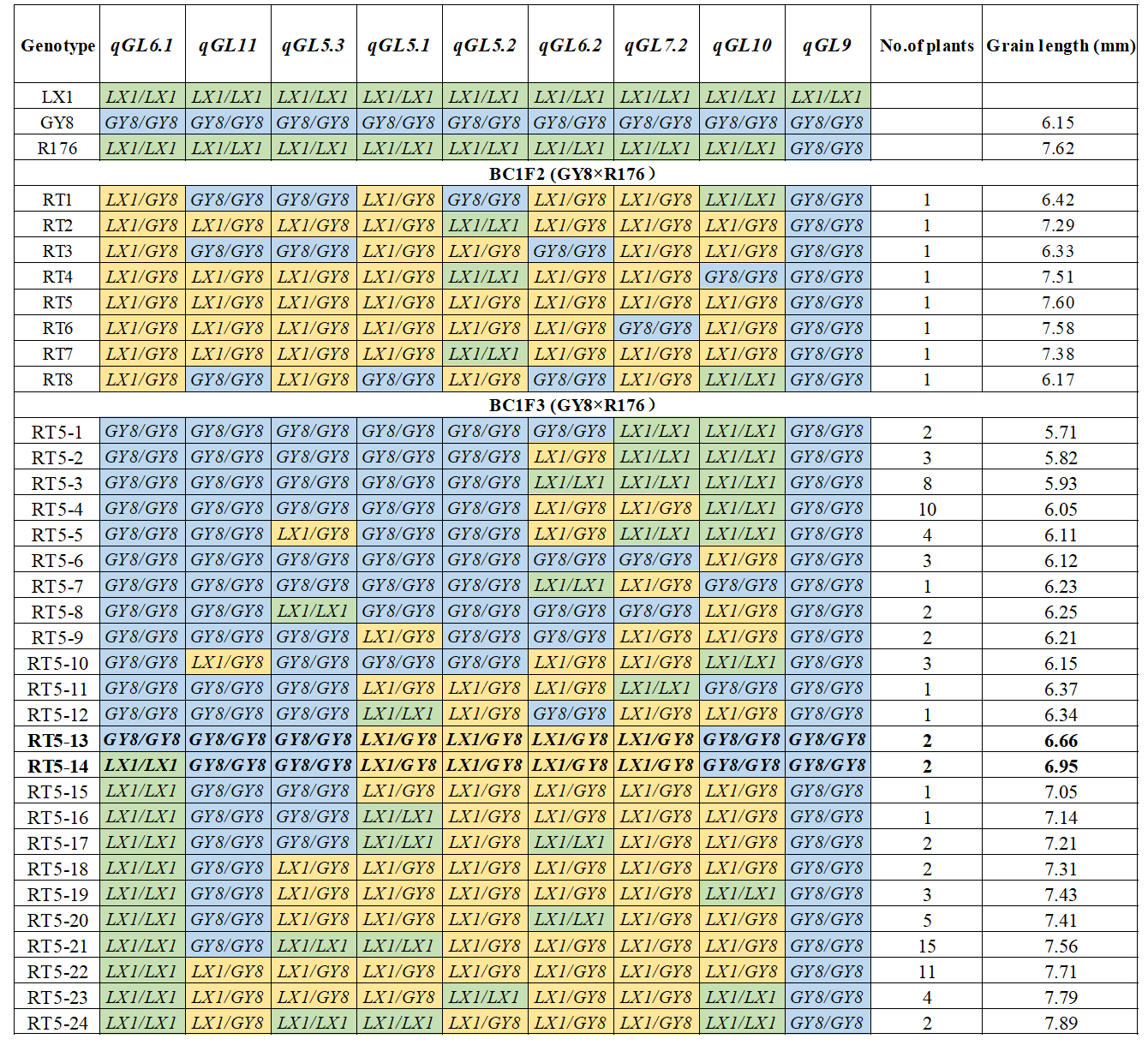

Supplement: Supplementary file 2 — Additional file 2. Fig. S1 The genotypic data at qGL11, qGL5.3, qGL5.1, qGL5.2, qGL6.2, qGL7.2 and qGL10 locus of some progenies derived from the heterozygous recombinants R5. [file 12284_2022_606_MOESM2_ESM.tif]

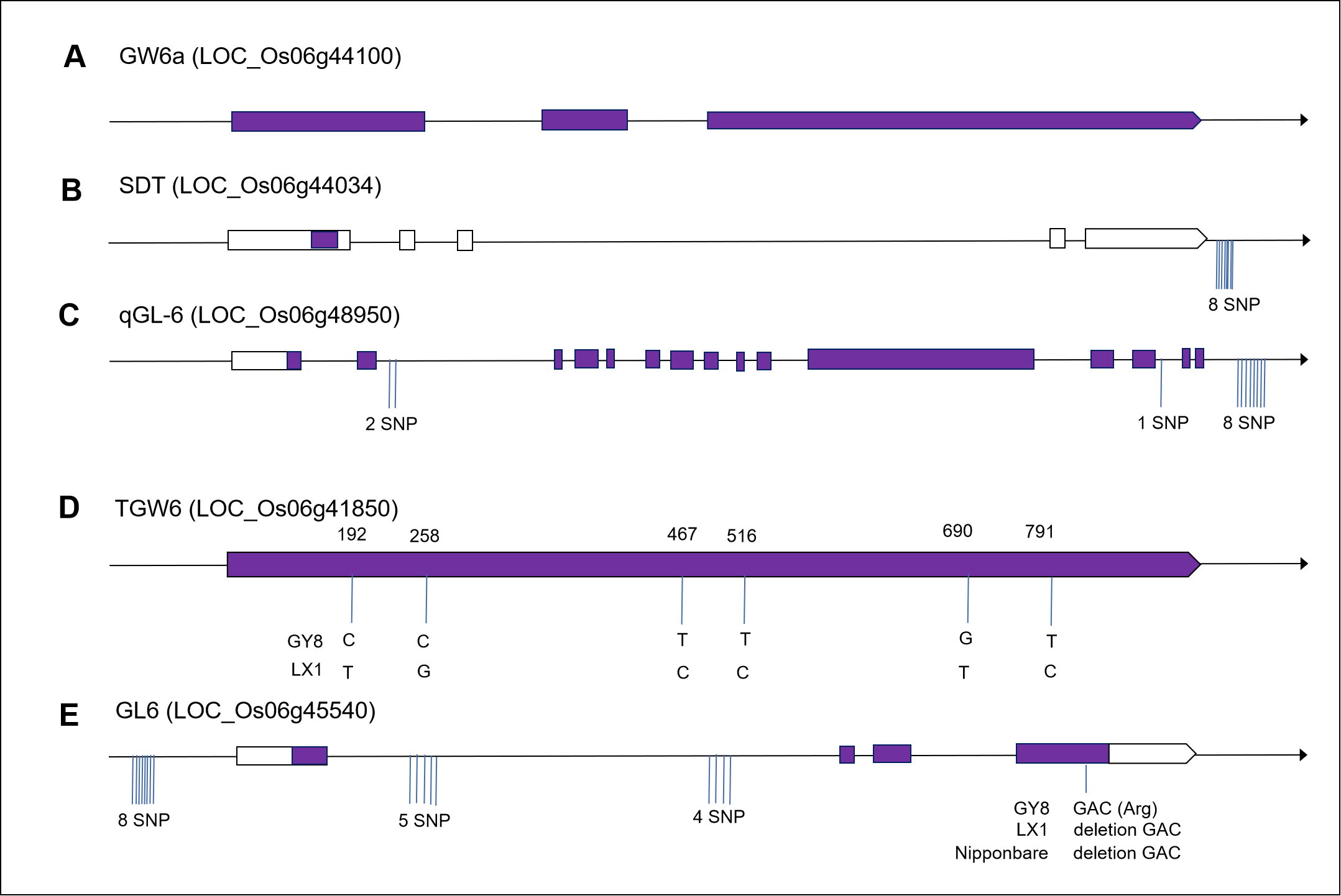

Supplement: Supplementary file 3 — Additional file 3. Fig. S2 Variations in the upstream, intron and downstream region of GW6a (A), SDT (B), qGL-6 (C), TGW6 (D) and GL6 (E) between Gangyuan8 (GY8) and Liaoxing1 (LX1). [file 12284_2022_606_MOESM3_ESM.tif]

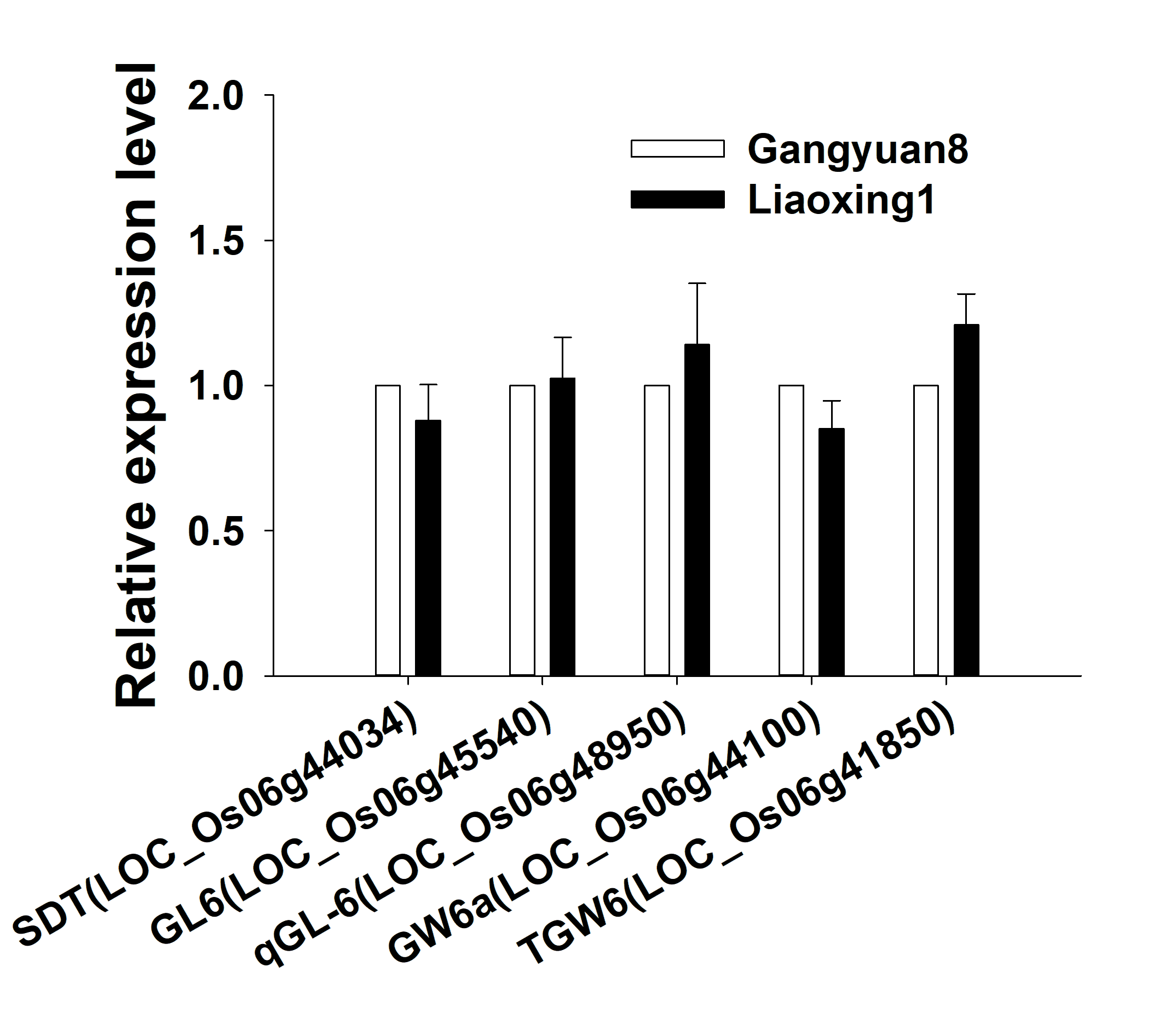

Supplement: Supplementary file 4 — Additional file 4. Fig. S3 The expression level of GW6a, SDT, qGL-6, TGW6 and GL6 by qPCR analysis. [file 12284_2022_606_MOESM4_ESM.tif]
